# Dr. Thomas H. Manley

**Published:** 1905-02

**Authors:** 


Dr. Thomas H. Manley, of New York, died at his home, January 13, 1905, aged 54 years. His death, which was caused by pneumonia, was somewhat sudden and came while he was in robust life and engaged in an active practice. He was visiting surgeon to the Harlem and Metropolitan hospitals.

